# Generation of ECG signals from a reaction-diffusion model spatially discretized

**DOI:** 10.1038/s41598-019-55448-5

**Published:** 2019-12-12

**Authors:** M. A. Quiroz-Juárez, O. Jiménez-Ramírez, R. Vázquez-Medina, V. Breña-Medina, J. L. Aragón, R. A. Barrio

**Affiliations:** 1 Instituto de Ciencias Nucleares, Universidad Nacional Autónoma de México. Circuito Exterior S/N, Ciudad Universitaria, 04510 Ciudad de México, México; 2 Instituto Politécnico Nacional, Escuela Superior de Ingeniería Mecánica y Eléctrica, Santa Ana 1000, San Francisco Culhuacán, 04430 Ciudad de México, México; 3 Instituto Polit écnico Nacional, Centro de Investigación en Ciencia Aplicada y Tecnología Avanzada, Cerro Blanco 141, Colinas del Cimatario, 76090 Querétaro, México; 4 Instituto Tecnológico Autónomo de México, Departamento Académico de Matemáticas, Rio Hondo 1, Col. Progreso Tizapán, 01080 Ciudad de México, México; 5 Centro de Física Aplicada y Tecnología Avanzada, Universidad Nacional Autónoma de México, Boulevard Juriquilla 3001, 76230 Querétaro, México; 6 Instituto de Física, Universidad Nacional Autónoma de México, Apartado Postal 20-364, 01000 Ciudad de México, México

**Keywords:** Applied mathematics, Software

## Abstract

We propose a model to generate electrocardiogram signals based on a discretized reaction-diffusion system to produce a set of three nonlinear oscillators that simulate the main pacemakers in the heart. The model reproduces electrocardiograms from healthy hearts and from patients suffering various well-known rhythm disorders. In particular, it is shown that under ventricular fibrillation, the electrocardiogram signal is chaotic and the transition from sinus rhythm to chaos is consistent with the Ruelle-Takens-Newhouse route to chaos, as experimental studies indicate. The proposed model constitutes a useful tool for research, medical education, and clinical testing purposes. An electronic device based on the model was built for these purposes

## Introduction

The human heart beats for about 2 to 3 billion times in a normal life. The heart pumping function is initiated by action potentials arising from the sinoatrial (*SA*) node. This electrical signal travels through the atrial muscles to the atrioventricular (*AV*) node. Finally, the electrical impulses propagate along the His-Purkinje complex causing the contraction of the ventricular muscles. This process leads to the blood supply into the whole body^1^. The depolarization and repolarization events occurring with each cardiac cycle reflects the ionic current flow which gives place to a time-varying electrical potential on the surface of the skin, which represents the physical information recorded in an electrocardiogram (ECG). Since the ECG signal contains information of the electrical events of cardiac cycle, it has been widely used for detecting abnormalities of the heart, usually called arrhythmias^1,2^. Among these is the particularly malignant ventricular fibrillation (VF) which without timely medical intervention causes the sudden cardiac death in a few minutes^3^. Since the VF is distinguished by a state of irregular arrhythmic contraction of the ventricular muscles, it has been signaled as a stochastic and erratic process. However, nonlinear dynamical systems theory is recognized as a useful tool that can contribute to the understanding of underlying mechanisms of lethal arrhythmias and cardiac diseases^4^.

Heart functioning can be modeled with different levels of detail. Some models describe the function of the heart at cell level^5,6^. Others include spatiotemporal characteristics of the heart rhythms^7,8^. There are also models based on ECG signals that describe the electrical activity of the heart at a macroscopic level^9,10^. In this realm, a relatively old idea is to consider the heart as a network of relaxation oscillators, following the pioneering studies developed by Van der Pol and Van der Mark (VdP)^11^. Since the work of VdP several modifications have been proposed to model the electrical activity in the heart because the VdP oscillator displays many features occurring in the biological context^12^. All models that reproduce the electrical activity of the heart using coupled nonlinear oscillators are complex and based on modified VdP coupled oscillators. Furthermore, the couplings usually include time delays, which can cause drastic changes in the system dynamics, and even the emergence of deterministic chaos^13^. Several studies about the effect of conduction delays in coupled oscillators have been reported^14^. The classical way to model the electrical activity of the heart is by using reaction-diffusion (RD) equations (see for example, refs. ^15,16^), because the propagation of electrical waves through the heart is similar to the propagation of nonlinear waves. In other systems, we could say that models based on VdP nonlinear oscillators are somewhat simplified and do not capture all the features one wants to model.

Experimental studies based on the analysis of electrocardiogram recordings of dog hearts^17–19^, which were electrically induced to a VF, suggest the presence of chaos, resulting from nonlinear deterministic dynamics^20^. Even more, in ref. ^21^. the fibrillation was studied in three stable biological models: chronic human atrial fibrillation, stabilized canine ventricular fibrillation and fibrillation like activity in thin sheets of canine and human ventricular tissue *in vitro*. By analyzing the experimental data recorded, the results indicate that fibrillation is an example of spatio-temporal chaos arising from a sinus rhythm following the Ruelle-Takens-Newhouse route to chaos^22^. In a previous paper^23^, we proposed an extension of the model developed in^24^ (which reproduces clinically comparable ECG waveforms) to produce chaotic responses associated with VF by the inclusion of an ectopic pacemaker (EP) that stimulates the ventricular muscles. The idea that the VF presents chaotic behavior may suggest new therapeutic strategies to avoid it.

In this work we propose a mathematical model based on a reaction-diffusion mechanism^25^ to simulate the generation of ECG signals.The model is discretized in a way that a set of three coupled nonlinear oscillators (not VdP type) is obtained and used to describe the electrical activity of the heart. The three nonlinear oscillators can be assigned to the natural pacemakers in the heart, and the ECG signals can be simulated as a combined signal of the variables of these oscillators. Although the present work and previous study^23^ were born from different inspirations, they maintain three common aspects: (1) are based on coupled nonlinear oscillators (the natural pacemakers in the heart are described by three coupled oscillators); (2) follow the same route to chaos; and (3) are capable to reproduce electrocardiogram signals of healthy hearts as well as some arrhythmias.

## Methods

### The model

Reaction-diffusion equations have been used to model the electrical activity of the heart^26,27^. Electric activation propagating along nerve fibers arise from the flow of ions across the cell membrane (ionic currents), that is, from the movement of ions inside or outside of the extracellular space^28^. These ionic currents are represented in the reaction kinetics added the diffusion equation for the membrane potential.

The Barrio-Varea-Aragon-Maini (BVAM) model^25^ is a generic reaction-diffusion system obtained by assuming mass conservation of two morphogens and by a Taylor expansion around an equilibrium point, retaining up to cubic non-linearities. This model has been used for describing a wide diversity of patterns present in biological or chemical systems^29,30^. The BVAM model exhibits extremely rich dynamics, ranging from Turing patterns, traveling waves, temporal oscillations and chaotic behavior^31^. In dimensionless form, the BVAM model is:

1a $$\frac{\partial u}{\partial t}=D{\nabla }^{2}u+\eta (u+av-Cuv-u{v}^{2}),$$

1b $$\frac{\partial v}{\partial t}={\nabla }^{2}v+\eta (bv+Hu+Cuv+u{v}^{2}),$$

where *u*(*x*, *t*) and *v*(*x*, *t*) describe two interacting variables at position *x* and time *t* with constant diffusion coefficient ratio *D*. These variables could be interpreted as chemical substances, morphogens or any other measurable quantity, depending on the particular system to be modeled. Here, *η*, *a*, *b*, *C* and *H* are parameters of the system. Zero flux (Newmann) boundary conditions are usually used. From a mathematical point of view, this election is justified due to the interest in self-organized patters and zero flux imply no external input. From a physiological point of view, zero flux imply electrical insulating boundaries.

As in^31^, we consider parameter values: *η* = 1, *a* = −1 and *b* = −3. These values situate the model in a region where it produces nonlinear oscillations in an excitable medium, and complies with the basic requirements to model the cardiac beat.

**Figure 1:**
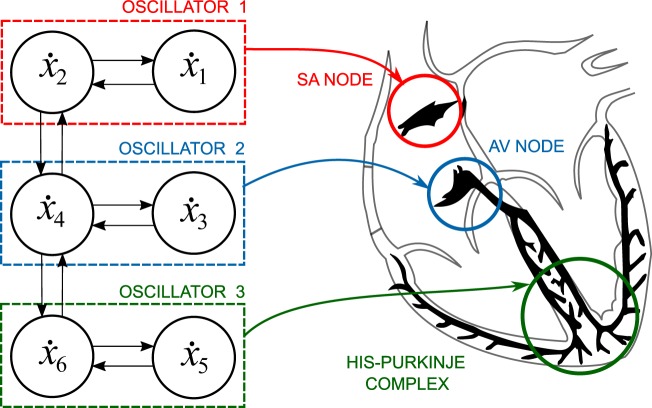
Diagram relating the cardiac natural pacemakers to the nonlinear variables *x*_1_ − *x*_6_ of the discretized BVAM model (2). Here, the action potentials of the SA node, AV node, and His-Purkinje complex are represented by coupled three oscillators, respectively.

Upon applying the simple Euler description to a discrete Laplacian, we recast Eq. (1) as a system of ordinary differential equations (ODE). In doing so, we associate the resulting system of differential equations to the natural pacemakers in the heart by taking into account a three-node standard stencil of the domain of length *L*. In this way, two nodes correspond to the boundaries *x* = 0, *x* = *L* and another one within the domain. As zero-flux boundary conditions are into consideration, *i.e. u*_*x*_ = *v*_*x*_ = 0 at *x* = 0, *L*, first derivatives are approximated by forward and backward differences. From this, we obtain *u*_4_ = *u*_3_, *v*_4_ = *v*_3_, *u*_0_ = *u*_1_ and *v*_0_ = *v*_1_. Upon defining *x*_1_ = *u*_1_, *x*_2_ = *v*_1_, *x*_3_ = *u*_2_, *x*_4_ = *v*_2_, *x*_5_ = *u*_3_ and *x*_6_ = *v*_3_, we obtain a six-component ODE system:

2 $$\begin{array}{rcl}{\dot{x}}_{1} & = & {x}_{1}-{x}_{2}-C{x}_{1}{x}_{2}-{x}_{1}{{x}_{2}}^{2},\\ {\dot{x}}_{2} & = & H{x}_{1}-3{x}_{2}+C{x}_{1}{x}_{2}+{x}_{1}{{x}_{2}}^{2}+\beta ({x}_{4}-{x}_{2}),\\ {\dot{x}}_{3} & = & {x}_{3}-{x}_{4}-C{x}_{3}{x}_{4}-{x}_{3}{{x}_{4}}^{2},\\ {\dot{x}}_{4} & = & H{x}_{3}-3{x}_{4}+C{x}_{3}{x}_{4}+{x}_{3}{{x}_{4}}^{2}+\beta ({x}_{6}-2{x}_{4}+{x}_{2}),\\ {\dot{x}}_{5} & = & {x}_{5}-{x}_{6}-C{x}_{5}{x}_{6}-{x}_{5}{{x}_{6}}^{2},\\ {\dot{x}}_{6} & = & H{x}_{5}-3{x}_{6}+C{x}_{5}{x}_{6}+{x}_{5}{{x}_{6}}^{2}+\beta ({x}_{4}-{x}_{6}),\end{array}$$

where *β* = 1/(Δ*x*)^2^ is the coupling constant in the discrete Laplacian. These three identical oscillators, coupled in series, could be interpreted as the three natural pacemakers of the heart, namely, the SA node, the AV node and the His-Purkinje complex^1^, as depicted in Fig. 1. Note that the system represents each pacemaker with similar oscillators and that they are coupled in series.

From the numerical integration of system (2), we note that the variables *x*_1_, *x*_5_, and *x*_2_, *x*_6_, respectively, are not independent to each other due to symmetry. Thus, a further reduction of the system (2) can be made. The resulting equivalent system of equations are simultaneously satisfied as *x*_2_ = *x*_6_, which yields that *x*_1_ = *x*_5_. Finally, system (2) is reduced to the four-component ODE system:

3 $$\begin{array}{rcl}{\dot{x}}_{1} & = & {x}_{1}-{x}_{2}-C{x}_{1}{x}_{2}-{x}_{1}{{x}_{2}}^{2},\\ {\dot{x}}_{2} & = & H{x}_{1}-3{x}_{2}+C{x}_{1}{x}_{2}+{x}_{1}{{x}_{2}}^{2}+\beta ({x}_{4}-{x}_{2}),\\ {\dot{x}}_{3} & = & {x}_{3}-{x}_{4}-C{x}_{3}{x}_{4}-{x}_{3}{{x}_{4}}^{2},\\ {\dot{x}}_{4} & = & H{x}_{3}-3{x}_{4}+C{x}_{3}{x}_{4}+{x}_{3}{{x}_{4}}^{2}+2\beta ({x}_{2}-{x}_{4}).\end{array}$$

Since the ECG signal is a composition of waves coming from different areas of the heart with different magnitudes, it can be considered, as a first approximation, that the ECG signal is a linear mixing of the electrical activations of the main cardiac pacemakers with different strengths^32^. In this way, signals that closely resemble ECG signals can be generated by an adequate linear combination of the variables *x*_*i*_:

4 $$ECG(t)={\alpha }_{1}{x}_{1}+{\alpha }_{2}{x}_{2}+{\alpha }_{3}{x}_{3}+{\alpha }_{4}{x}_{4}\mathrm{}.$$

## Results

### Ventricular fibrillation

As it was already mentioned, experimental studies^21^ suggest that ventricular fibrillation is a form of spatio-temporal chaos that arises from a normal rhythm through the so-called Ruelle-Takens-Newhouse scenario^22^. In what follows we will show that system (3) can exhibit this behaviour under the variation of the control parameter *H*.

**Figure 2:**
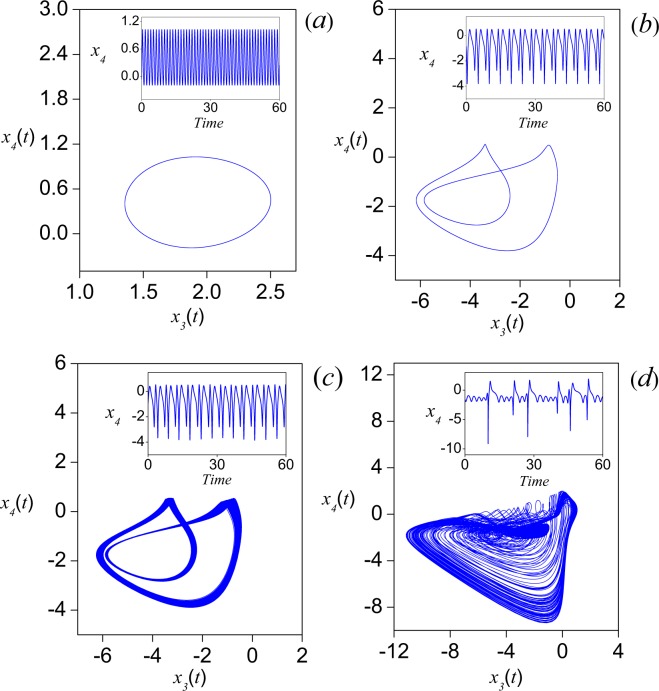
Phase portraits for four different values of the control parameter *H*, after *t* = 500 seconds. (**a**) *H* = 7, (**b**) *H* = 2.74, (**c**) *H* = 2.72972, and (**d**) *H* = 2.7126. In the insets, the corresponding time series (in seconds) for the *x*_4_ variable are shown. Numerical simulations were carried out with *C* = 1.35 and *β* = 4.

Since the analytical treatment of nonlinear terms is often prohibitive, five numerically calculated quantities are used to analyze the system, namely, time series, phase portrait, power spectrum, bifurcation diagram and largest Lyapunov exponent. First of all, we observe that *H* governs the linear terms, while *C* is the ratio of the strengths of quadratic and cubic nonlinearity. By making a linear analysis of the model (1), without the diffusion term (resulting equations have the same form that the equations of individual oscillators in the system (3)), the equilibrium point (0, 0) presents stationary solutions for $0 < C < \sqrt{2}$. In order to keep the behavior simple, we have fixed *C* = 1.35 and define *H* as the control parameter, in the interval [1, 20]. System (3) was solved numerically using the step-fixed Runge-Kutta method^33^ with Δ*t* = 0.005^34,35^. To boot up system (3), a perturbed state around the equilibrium point (0, 0, 0, 0) can be obtained by disturbing any of the dynamical variables. We consider the case **x**(0) = (0, 0, 0.1, 0) as one of the possible alternatives to stimulate the system. It is worth mentioning that all simulations in this work were carried out by using the same numerical method, integration step and initial conditions, as well as the parameter values *C* = 1.35 and *β* = 4. In Fig. 2, the phase portraits for *x*_4_ versus *x*_3_ are shown for different values of *H*. As depicted in Fig. 2(a), for *H* = 7 a limit cycle is formed. For *H* = 2.74, the limit cycle doubles its period (Fig. 2(b)) and for *H* = 2.72972, a torus is generated (Fig. 2(c)), which corresponds to a quasi-periodic oscillation where at least two incommensurate frequencies are involved (see the inset). When *H* = 2.7126 (Fig. 2(d)) a strange attractor seems to be formed, thus a completely irregular time series can be observed (inset).

**Figure 3:**
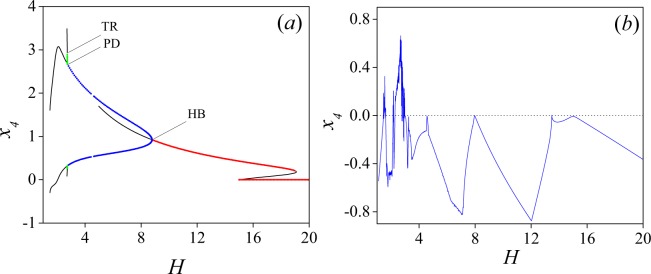
Numerically calculated bifurcation diagram of model (3) using AUTO. (**a**) Supercritical Hopf (HB), period doubling (PD), and torus (TR) bifurcations are detected at *H* = 8.779267, 2.742524 and 2.72972, respectively. Here, red line corresponds to a family of stationary states and blue line to a set of stable limit cycles. Periodic solutions with half the frequency are indicated by the green line. Finally, the loss of stability in the period doubling gives place to the quasi-periodicity (black line). Small perturbations transform the quasiperiodic orbits into a chaotic motion. (**b**) Largest Lyapunov exponents as *H* is varied.

**Figure 4:**
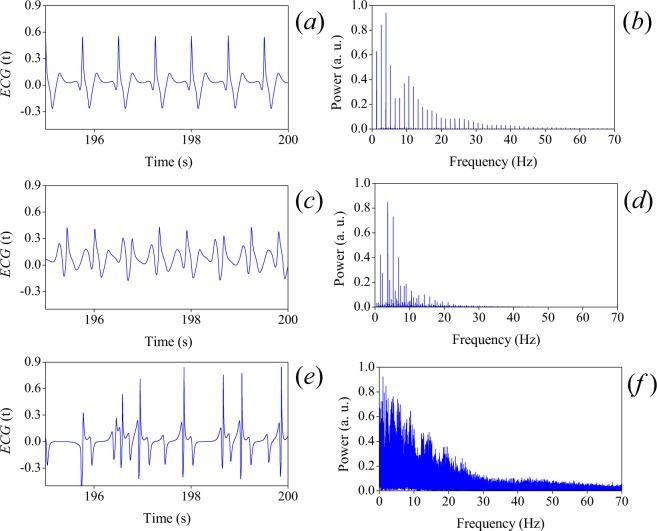
ECG’s corresponding to: (**a**) normal rhythm (*H* = 3), (**c**) quasiperiodicity (*H* = 2.729) and (**e**) VF (*H* = 2.164). Their corresponding power spectra, (**b**), (**d**) and (**f**), are shown on the right of each ECG plot, respectively.

To confirm the results suggested by the time series and the phase portraits (see also the power spectra shown in Fig. 4 below), we have numerically computed the bifurcation diagram using the software AUTO^36^, by varying the control parameter in the interval 1 ≤ *H* ≤ 20. The bifurcation diagram is shown in Fig. 3(a), where one could notice that steady-states undergo transitions from stationary to Hopf to Period Doubling to Two Torus to Three Torus (namely Chaos). As it seen in Fig. 3(a) all the interesting bifurcations occur in the interval [1, 9]. However, we decided to keep the results up to *H* = 20, to show the rich variety of dynamics of the system.

A family of stationary states is found for values of *H* ≥ 9. As *H* is decreased, a branch consisting of stable limit cycles arises from a supercritical Hopf bifurcation (HB) point at *H* = 8.779267. This branch loses stability as a Period Doubling (PD) point is attained at *H* = 2.742524. Finally, this branch undergoes a transition through a Torus point (TR) at *H* = 2.72972. Notice that these features are shown in Fig. 2, where the initial limit cycle is continuously shifted forming a torus, which gives place to quasi-periodicity. Finally, small perturbations transform the quasiperiodic orbit into a chaotic motion, or a strange attractor. All these results are consistent with the Ruelle-Takens-Newhouse route to chaos^22,37^. To confirm the existence of chaos, the largest Lyapunov exponents (*λ*) were numerically computed. Figure 3(b) shows the largest Lyapunov exponent as function of *H*, using an incremental step of 0.009 for *H*. Observe positive values in the region of chaos.

Since the model in Eq. (3) is written in non-dimensional form, we need to add a scale factor to obtain ECG signals with the correct oscillations in time. If a factor Γ_*t*_ multiplies the right hand side of (3), i.e., $\dot{{\bf{x}}}={\Gamma }_{t}\,f({\bf{x}},t)$, then Γ_*t*_ follows a linear dependence on the heart rate (in beats per minute), given by the following expression:

5 $${\Gamma }_{t}(H{R}_{bpm})=0.08804\,H{R}_{bpm}-0.06754,$$

where *HR*_*bpm*_ is the heart rate. To obtain above equation, the dependence of the scaling factor Γ_*t*_ with the heart rate was found by numerically integrating system (3), taking a normal ECG waveform as reference. Given this dependence, we fit it with a monomial function.

In Fig. 4 we show ECG’s corresponding to (*a*) normal rhythm (*H* = 3), (*c*) quasiperiodicity (*H* = 2.729) and (*e*) VF (*H* = 2.164), with their corresponding power spectra in the right hand side. For all rhythms in this subsection, the numerical simulations of (4) were carried out with *C* = 1.35, *β* = 4, and scaling coefficients: *α*_1_ = −0.024, *α*_2_ = 0.0216, *α*_3_ = −0.0012, and *α*_4_ = 0.12. *α*_*i*_ coefficients were calculated using a modification of the supervised learning algorithm called perceptron^38,39^. The time scaling factors, Γ_*t*_ = 7, for normal and period doubling rhythms, and Γ_*t*_ = 17 for VF were computed using (5), in order to recover the physiological times.

Some words concerning the role of the model parameters are in order: coefficients *α*_*i*_ correspond to the contribution of each compartment *x*_*i*_ (related to nodes). *β* captures the coupling between nodes, considering that it is a dynamical network. *H* and *C* control the compartment-to-compartment local interaction of the dynamical network. Finally, the time-scale parameter Γ_*t*_ can be justified in the same way as the parameter *C*_*m*_ used to correct the time scale of the action potential in the Purkinje fibers in Noble’s model^40^ and the parameters *χ* and *C*_*m*_ in Sundnes *et al*.^41^ (Sundnes’ model).

### Numerical simulations

**Figure 5:**
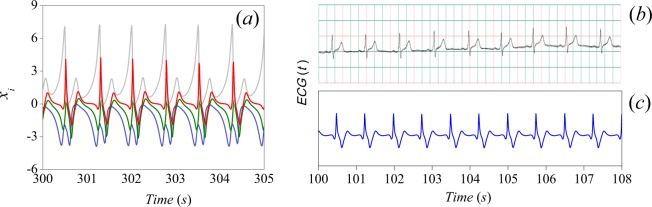
Time series: (**a**) The *x*_*i*_ variables of (3): *i* = 1 (blue line), *i* = 2 (green line), *i* = 3 (gray line) and *i* = 4 (red line). (**b**) Real ECG^42^. (**c**) ECG signal obtained from (4).

Using (4) the ECG signal corresponding to the normal sinus rhythm is obtained with the following parameter values: *α*_1_ = −0.024, *α*_2_ = 0.0216, *α*_3_ = −0.0012, *α*_4_ = 0.12 and Γ_*t*_ = 7. In Fig. 5 the ECG signal (standard Einthoven lead II) and the *x*_*i*_ variables, obtained by numerical simulation of Eqs. (4) and (3), are shown. In the figure we also show the simulated ECG (*c*) to be compared with an ECG signal obtained from ref. ^42^ (shown in (*b*)). Our model reproduces only lead II, which forms part of the main leads in Einthoven’s triangle and is commonly used by cardiologists to provide a rhythm strip^43^.

The time evolution of the state variables in (3) is shown in Fig. 5(a). Notice that *x*_4_ alone generates a signal closely related to the ECG waveform. This is because *x*_4_ contains the dynamics of the HP complex and the AV node combined, as a result of the reduction from the system of Eqs. (2) to (3), that is, from three to two oscillators. However, we have proposed the linear combination (4) in order to obtain a better approximation, as it is shown in Fig. 5(c), from where we observe that the model captures the most important characteristics of a real ECG. Notice also that the only coupling of the oscillators is through parameter *β*, which plays a crucial role on modifying the output waveform.

**Figure 6:**
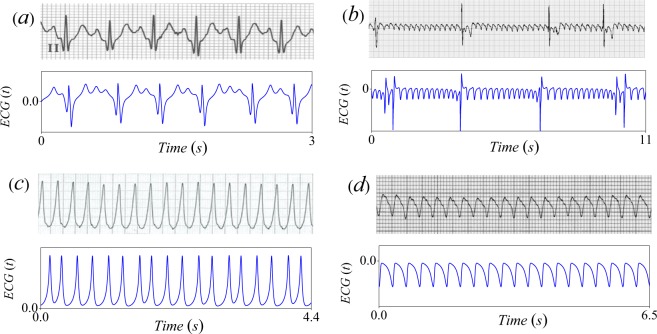
Comparison of ECG plots obtained from experimental observations (top panels) and the reduced system (3) (bottom panels). (**a**) Sinus tachycardia^44^, (**b**) Atrial flutter^42^, (**c**) Ventricular tachycardia^45^ and (**d**) Ventricular flutter^46^.

The parameter space was explored to reproduce the most important pathological arrythmias (see Table 1). Here, parameter *H* was selected from the bifurcation diagram and *α*_*i*_ coefficients in Table 1 were calculated using the modified perceptron algorithm like in the above subsection. Our results are exhibited in Fig. 6, where they are compared with ECG (standard Einthoven lead II) obtained from^42,44–46^.

**Table 1:** Parameters for arrhythmias. In all cases *C* = 1.35 and *H* = 2.848.

Pathology	Parameters
Sinus Tachycardia	*H* = 2.848, *α*_1_ = 0, *α*_2_ = −0.1, *α*_3_ = 0 *α*_4_ = 0 and Γ_*t*_ = 21.
Atrial Flutter	*H* = 1.52, *α*_1_ = −0.068, *α*_2_ = 0.028, *α*_3_ = −0.024, *α*_4_ = 0.12 and Γ_*t*_ = 13
Ventricular Tachycardia	*H* = 2.178, *α*_1_ = 0, *α*_2_ = 0, *α*_3_ = 0 *α*_4_ = −0.1 and Γ_*t*_ = 21
Ventricular Flutter	*H* = 2.178, *α*_1_ = 0.1, *α*_2_ = −0.02, *α*_3_ = −0.01, *α*_4_ = 0 and Γ_*t*_ = 13.

In sinus tachycardia (Fig. 6(a)) an exaggerated acceleration of heart rate greater than 100 bpm occurs. In Fig. 6(b) an atrial flutter can be observed. Ventricular tachycardia (VT) is a fast rhythm that begins in the ventricles. It is characterized by a pulse greater than 100 bpm (see Fig. 6(c)). Finally, in Fig. 6(d), we show a ventricular flutter, which is characterized by a very rapid (150 up to 250 bpm) and regular ectopic ventricular rhythm. It is usually preceded by VT and this condition is fatal if untreated. Notice the striking similarities between the theoretical results and all pathological ECG. An important feature of our model is that it is capable to reproduce several well-known rhythm disorders with a relatively good matching of the shapes and amplitudes of realistic ECGs.

### Electronic implementation

**Figure 7:**
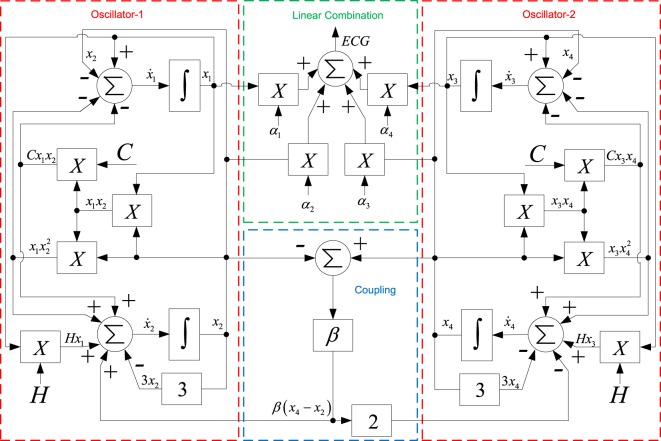
Block diagram of the system (3) and the linear combination (4).

The system (3) can be electronically implemented by using analog multipliers and inverting configurations of operational amplifiers (OPAMPs), such as the adder, integrator, and gain. In other words, we use the working principle of an analog computer to represent physical variables of the mathematical model through input and output voltage variables of an analog electronic circuit. In this way, in order to simulate any mathematical model in an analog computer, the sequence of mathematical operations involved in the process must be described by means of functional block diagrams. Figure 7 shows the block diagram of the system (3) and the linear combination in (4) to obtain the ECG signal.

Each mathematical operation involved in the block diagram can be electronically implemented with linear and nonlinear electronic components. Note that the block diagram obtained for *x*_1_ and *x*_2_ (named Oscillator-1) is equal as that obtained with *x*_3_ and *x*_4_ (named Oscillator-2). Therefore, both oscillators can be implemented with identical electronic circuits.

**Figure 8:**
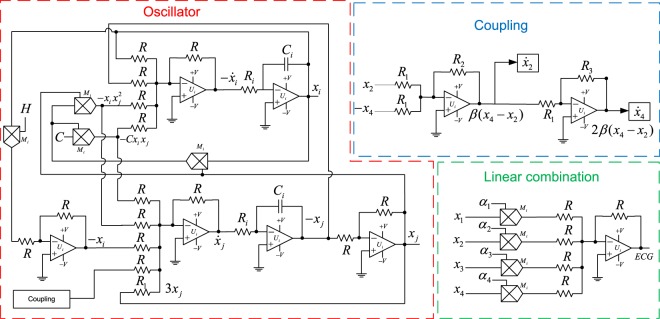
Proposed analog electronic circuit.

Figure 8 shows the synthesized analog circuit for an oscillator, the coupling and the linear combination (4). Since inverting configurations of OPAMPs are used, an inherent sign inversion must be taken into account in the design. *R*_*i*_, *C*_*i*_, *U*_*i*_ and *M*_*i*_ stand for resistors, capacitors, general purpose operational amplifier and analog multipliers, respectively. The values of *R*, *R*_1_, *R*_2_, *R*_3_, *R*_*i*_, *C*_*i*_ are defined by the parameters of the system. They must satisfy:

6 $$\frac{R}{{R}_{1}}=\mathrm{3,}\,\frac{{R}_{2}}{{R}_{1}}=\beta ,\,\frac{{R}_{3}}{{R}_{1}}=\mathrm{2,}\,\frac{1}{{R}_{i}{C}_{i}}={\Gamma }_{t}$$

We calculated the resistors and capacitors involved in Fig. 8, considering Γ_*t*_ = 7 and *β* = 4. By selecting *R* = 3 *K*Ω, *R*_1_ = 1 *K*Ω, *R*_2_ = 4 *K*Ω, *R*_3_ = 2 *K*Ω, *R*_*i*_ = 14 *k*Ω, *C*_*i*_ = 10 *μF*, the conditions given by (6) are satisfied. *U*_*i*_ and *M*_*i*_ correspond to OPAMPs and analog multipliers of the series MC1458 and AD633, respectively.

An important feature of the proposed electronic implementation is that the parameters *H*, *C* and *α*_*i*_ are introduced in the circuit via a voltage signal. This is particularly relevant because the value of each parameter can be defined independently from the circuit, this implies that in order to set the desired parameter value it is not required to physically change some electronic components (resistors or capacitors).

**Figure 9:**
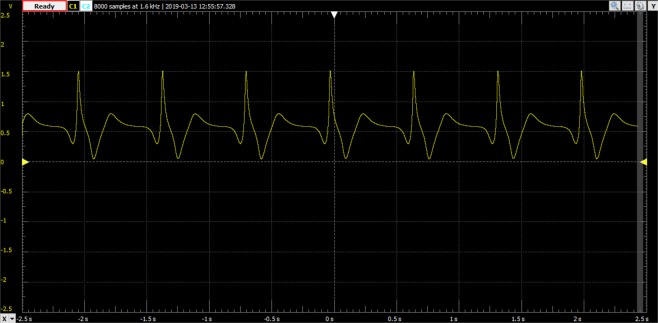
Oscilloscope output of the proposed analog electronic circuits for the normal sinus rhythm.

As an example, in Fig. 9, the output of the proposed analog electronic circuits is shown for the normal sinus rhythm, for the same parameter values used in Fig. 5(c).

## Discussion

In this work a model to generate electrocardiogram signals by means of coupled nonlinear oscillators was proposed. By defining a linear combination of the contributions of the oscillator variables involved, healthy ECG signals are obtained, as well as realistic ECG’s presenting several crucial arrhythmias.

An important feature of our model is that the transition from normal rhythm to VF is controlled by a single parameter, contrary to other models where several parameters should be varied or an external oscillator or signal must be included to reproduce chaotic behaviours associated with VF^23,32^. Here, the control parameter *H* plays a boosting role from variable *u* to *v*; this role can also be seen in the reduced system (3).

We considered the case of ventricular fibrillation in more detail. With the aid of numerical tools, we conclude that in our model, the ECG of this dangerous arrhythmia is a chaotic signal and that the transition from sinus rhythm to VF, when varying a control parameter, follows the Ruelle-Takens-Newhouse route to chaos.

Finally, as the proposed model constitutes a useful tool for medical education, research and testing purposes, an electronic implementation of the model was proposed and built. Observe that an experimented physician could object that our theoretical ECG’s do not exactly resemble the real ones from everyday patients. This points out to the limitations of any modeling, which necessarily represents a simplification of the real world. Nevertheless, our model captures the main features of the normal and abnormal recordings and opens new ways to the understanding of the origins of the most usual heart failures.
